# Inflammatory Markers and Haptoglobin Polymorphism in Saudi with Non-insulin-dependent Diabetes Mellitus

**DOI:** 10.5539/gjhs.v5n1p135

**Published:** 2012-11-11

**Authors:** Abdelmarouf Mohieldein, Mohammad Alzohairy, Marghoob Hasan, Amjad A. Khan

**Affiliations:** 1 Department of Medical Laboratories, College of Applied Medical Sciences, Qassim University, Buraidah, Saudi Arabia; 2 Department of Optometry, College of Applied Medical Sciences, Qassim University, Buraidah, Saudi Arabia

**Keywords:** C-reactive protein, interleukin-6, haptoglobin, acute phase proteins, haptoglobin phenotypes, type 2 diabetes, non-insulin-dependent diabetes mellitus

## Abstract

**Objectives:**

Haptoglobin (Hp) polymorphism associated with clinical evolution of several inflammatory diseases and considered as a predictive factor for development of diabetes complications. We designed the present study to investigate the frequency distribution of Hp phenotypes among Saudi with non-insulin-dependent diabetes mellitus compared to healthy nondiabetic subjects. Moreover, we explored the possibility of relationship between serum levels of inflammatory markers (namely, high-sensitive C-reactive proteins “*hs*-CRP”, interleukin (IL)-6, and Hp) and Hp phenotypes.

**Methods:**

In the present case-control study, we enrolled 60 type 2 diabetic patients as the study group and 60 healthy subjects as the control group. We assayed serum levels of Hp and *hs*-CRP by immunoturbidimetric method; while IL-6 was measured by ELISA. Native polyacrylamide gel electrophoresis was used for determination of Hp phenotypes.

**Results:**

In type 2 diabetics, serum concentrations of IL-6, *hs*-CRP, and Hp were significantly elevated and correlated to body mass index. Moreover, there was a significant correlation between plasma glucose level and Hp (r = 0.577, p = 0.000), IL-6 (r = 0.448, p = 0.000), and *hs*-CRP (r = 0.380, p = 0.001). In addition, data demonstrated a positive correlation between HbA1c and Hp (r = 0.521, p = 0.000), IL-6 (r = 0.420, p = 0.001), and *hs*-CRP (r = 0.353, p = 0.008). Hp 2-1 phenotype predominated among subjects in both study and control groups. No significant association between Hp phenotypes with any of the investigated inflammatory markers was documented.

**Conclusion:**

Inflammation may represent the link between type 2 diabetes and obesity. Hp 2-1 was the predominant phenotype among Saudi type 2 diabetics as well as healthy subjects. In addition to Hp; other possible genetic polymorphisms like CRP may have its effect on diabetes through different mechanisms.

## 1. Introduction

Diabetes Mellitus (DM) is one of the main threats to human health in this century (Schwarz et al., 2007). Expert Committee proposed two major classes of DM and named them, insulin-dependent diabetes mellitus (IDDM, type 1 diabetes) and non-insulin-dependent diabetes mellitus (NIDDM, type 2 diabetes) (2002). In Saudi Arabia; DM has become more apparent in the last two decades as a result of dramatic changes in the Saudi population lifestyle typified by unhealthy dietary habits that rich in both sugars and fat, sedentary lifestyle, and high rates of obesity (Mohieldein et al., 2011).

Insulin resistance (IR) and progressive pancreatic beta cell failure are key factors in the development of non-insulin-dependent diabetes mellitus (Maiese et al., 2010). Inflammation has been hypothesized to be associated with IR and precede the development of clinically overt diabetes (Pradhan et al., 2002). Moreover, the possession of a particular phenotype may offer some protection against the development of a variety of common disorders including diabetes (Dasgupta et al., 2008).

Haptoglobin (Hp), a hepatocyte derived serum α-2-Sialoglycoprotein, is a positive acute phase reactant (Wobeto et al., 2009; Mishra et al., 2010). In man, there are two common alleles for Hp denoted 1 and 2, and correspondingly, three different possible phenotypes (Hp1-1, Hp2-1, and Hp2-2) which have significant structural and functional differences (Vitalis et al., 2011). The Hp 2 allele protein product appears to be an inferior antioxidant and anti-inflammatory compared with the Hp 1 allele protein product. Moreover, Hp 1 is more efficient both in preventing heme release from the Hp-Hb complexes and in promoting uptake by the CD163 macrophage receptor (Quaye, 2008; Costacou et al., 2008; A´lvarez-Blasco et al., 2009). Hp polymorphism has been demonstrated to be associated with the prevalence and clinical evolution of many inflammatory diseases (Delanghe & Langlois, 2002). In addition, it has been demonstrated that Hp polymorphism is predictive of the development of various microvascular and macrovascular complications of diabetes (Burbea et al., 2004).

To our knowledge, there is no study conducted to determine the genetic predisposition of Saudi type 2 diabetics according to Hp polymorphism. We designed the present study to investigate the frequency distribution of Hp phenotypes among Saudi with non-insulin-dependent diabetes mellitus compared to healthy nondiabetic subjects. Moreover, a possible relationship between serum levels of inflammatory markers (high-sensitive C-reactive proteins “*hs*-CRP”, interleukin (IL)-6, Hp) and Hp phenotypes was explored.

## 2. Patients & Methods

### 2.1 Study Design & Subjects

This case - control study was conducted on a group of type 2 diabetic patients (n=60; 22 males, 38 females) attended at the diabetes clinic, King Fahd Specialist Hospital, Qassim. Diabetes was defined by fasting blood glucose ≥7.0 mmol/L (126 mg/dL), the use of hypoglycemic agents, or both. Sixty apparently healthy subjects were recruited from public places to represent the control group. They were neither had been diagnosed as having diabetes nor use hypoglycaemic medication.

### 2.2 Blood Sampling

Venous blood sample collected from each subject after informed consent in one heparinized vacutainer (4 ml) and one plain vacutainer (4 ml) to obtain plasma and serum respectively. All blood tubes were maintained at 4°C during transportation to the laboratory. After centrifugation at 3000 rpm for 15 min, aliquots of plasma and serum were stored at -80°C until analysis.

### 2.3 Hp Phenotyping

Hp phenotyping was performed by little modification of the method Linke as we described elsewhere (Hasan et al., 2012). Briefly; using a protein vertical mini-gel electrophoresis system (Bio-Rad mini protean III device; USA), total polyacrylamide concentrations of 7.0% and 4.0% were prepared respectively for separation and stacking gels of native-PAGE. Hp phenotype bands (Wursters blue) were identified by staining with TMPD (N, N.N’.N’-tetramethylphenylenediamine).

### 2.4 hs-CRP & Hp Assay

Serum concentrations of *hs*-CRP and Hp were assayed by a latex immunoturbidimetric method using Turbi Quick analyzer (vital Diagnostics, Italy). The linearity limits up to 5 mg/L and up to 250 mg/dL for *hs*-CRP and Hp respectively. Analysis was done according to manufacturer instructions.

### 2.5 IL-6 Assay

A quantitative sandwich enzyme-linked immunosorbent assay (ELISA) method was performed to determine serum IL-6 concentrations using commercial kits purchased from USCN LIFE, China. Calibration curve was prepared from standards with concentrations of 0.156, 0.312, 0.625, 1.25, 2.5, 5.0, 10.0 ng/ml. The color change was measured spectrophotometrically at 450 nm using bioMerieux Reader 250 version 2.0.5.

### 2.6 Estimation of Blood Glucose and HbA1c

Plasma glucose levels were determined by end-point enzymatic method (Glucose oxidase-glucose peroxidase) using kits manufactured by Human Diagnostics, Wiesbaden, Germany.

Glycated haemoglobin (HbA1c) was measured from whole blood by Latex immunoturbidimetric method using commercially available kit supplied by Vital Diagnostic, Italy.

### 2.7 Measurement of BMI

Body weight and height were recorded for each subject. Body mass index (BMI) was calculated as weight (in kilograms) divided by height (in metres) squared. The WHO classification for BMI was used to determine the degree of obesity (World Health Organ Tech Rep Ser, 1995). Subjects were categorized as normal if BMI was less than 25 kg/m^2^ overweight if BMI was between 25-29.9 kg/m^2^, and obese if BMI was greater than or equal to 30 kg/m^2^.

### 2.8 Statistical Analysis

The data analyzed using the statistical package for social sciences (SPSS) software (version 13, Chicago, IL, USA). Results expressed as mean ±SD or number (percent) where appropriate. Comparison of continuous variables between patients and control subjects was performed with student t-test and *p* values < 0.05 considered as statistically significant. Pearson`s correlation was used to test if the examined inflammatory markers (Hp, IL-6, *hs*-CRP) were correlated with age, blood glucose, and HbA1c. To detect whether Hp phenotypes distribution was in agreement with Hardy-Weinberg equilibrium; we determined Chi-square test. Values of p > 0.05 were considered to be in Hardy-Weinberg equilibrium.

### 2.9 Ethical Consideration

The protocol of this study was approved by the institutional review board of Deanship of Scientific Research, Qassim University, Saudi Arabia. Participation was voluntary and verbal consent was acquired from each participant prior to sample collection. Confidentiality of all participants was maintained as no names were requested.

## 3. Results

### 3.1 Characteristics of the Study Participants

This study recruited 60 type 2 diabetics (study group) and 60 healthy nondiabetic subjects (control group). Most of the participants in both groups were overweight or obese. The patients characterized by significantly (p= 0.000) higher blood glucose and glycated haemoglobin levels compared to controls. The demographic and clinical characteristics of all subjects are summarized in Table 1.

**Table 1:** Demographic and clinical characteristics of the study participants, comparing type 2 diabetic patients to healthy (nondiabetic) subjects

Variable	Patients n=60	Controls n=60	p-value
Gender (male /female)	22/38	31/29	.098
Age (yr)	50.59±9.2	45.98±9.9	.013*
Weight (kg)	80.3±17.4	75.1±15.2	.083
Height (cm)	160.9±5.5	159.7±14.6	.572
BMI (kg/m^2^)	31.0±6.8	29.6±6.4	.227
Blood Glucose (mmol/l)	10.9±4.4	5.5±1.9	.000*
HbA1c (%)	8.5±2.1	5.0±1.4	.000*
Duration of DM (years)	9.53±6.2	----	……

Abbreviations: BMI, body mass index; DM, diabetes mellitus; HbA1c, glycated haemoglobin Data presented as mean ± SD for all variables

* p-value <0.05; compared type 2 diabetic patients to nondiabetic healthy subjects

### 3.2 Markers of Inflammation

Serum levels of the inflammatory markers (IL-6, *hs*-CRP, Hp) were significantly increased (p = .000, .019, and .000 respectively) in type 2 diabetics compared to control subjects. Moreover, the increase in the concentrations of the aforementioned inflammatory markers (*hs*-CRP and Hp) was BMI dependent as shown in Tables 2 & 3.

**Table 2:** Serum levels of IL-6, *hs-*CRP, and Hp in types 2 diabetic patients compared to healthy control subjects (Mean ± SD)

analyte	Patients (n=60)	Controls (n=60)	*p*-value
IL-6 (ng/ml)	7.855±1.2	4.955±1.7	.000*
*hs*-CRP (mg/l)	2.09±0.8	1.70±0.7	.015*
Hp (mg/dl)	120.15±14.7	84.72±13.0	.000*

Abbreviations: IL-6: interleukin 6; *hs*-CRP: high sensitive C-reactive protein; Hp: haptoglobin.

* P-value <0.05; p value compared diabetic patients to healthy nondiabetic controls

**Table 3:** Serum levels of inflammatory markers (Hp, *hs*-CRP, and IL-6) in types 2 diabetic patients related to different BMI categories (Mean ± SD)

BMI (kg/m^2^)	Hp (mg/dL)	*hs*-CRP (mg/L)	IL-6 (ng/mL)
< 25 (n=15)	108.92±17.4	1.31±0.242	7.06±0.41
25 – 29.9 (n=22)	120.83±12.1 (p^1^= 0.038*)	2.15±0.63 (p^1^= 0.004*)	7.80±1.17 (p^1^= 0.250)
≥ 30 (n=23)	122.74±18.6 (p^2^= 0.036*)	2.26±0.85 (p^2^= 0.007*)	8.05±1.2 (p^2^= 0.134)

Abbreviations: IL-6: interleukin 6; *hs*-CRP: high sensitive C-reactive protein; Hp: haptoglobin; BMI, body mass index

* P-value < 0.05;

p1 value compared the inflammatory marker in diabetic patients with BMI category (25-29.9) to those diabetics with BMI less than 25 p2 value compared inflammatory marker in diabetic patients with BMI category (≥30) to those diabetics with BMI less than 25

### 3.3 Relationship between Inflammatory Markers and Selected Variables (age, blood glucose, and HbA1c) in Type 2 Diabetics

A positive correlation was found between blood glucose and levels of *hs*-CRP (r = 0.380, P = .001), Hp (r = 0.577, P = .000), and IL-6 (r = 0.448, P = .000). Moreover, Pearson’s correlation analyses showed significant associations of HbA1c with levels of *hs*-CRP (r = 0.353, P = .008), Hp (r = 0.521, P = .000), and IL-6 (r = 0.420, P = .001). Although of a positive correlation demonstrated between age and *hs*-CRP (r = 0.226, P = .043), there was no significant association between age and Hp or Il-6 (p> 0.05). The set of correlation coefficients and P-values for the inflammatory markers, age, blood glucose, and HbA1c is shown in Table 4.

**Table 4:** The correlations between inflammatory markers (Hp, IL-6, *hs*-CRP) and selected variables (age, Blood Glucose, and HbA1c) in type 2 diabetics

	Hp (mg/dl)		IL-6 (ng/ml)		*hs*-CRP (mg/l)	
Age	*r= 0.200*	*p= .069*	*r= 0.115*	*p=.376*	*r= 0.226*	p=.043*
Blood glucose	*r= 0.577*	p= .000*	*r= 0.448*	p= .000*	*r= 0.380*	p= .001*
HbA_1c_	*r= 0.521*	p= .000*	*r= 0.420*	p= .001*	*r= 0.353*	p= .008*

Abbreviations: IL-6: interleukin 6; *hs*-CRP: high sensitive C-reactive protein; Hp: haptoglobin; HbA1c: Glycated haemoglobin

* P-value < 0.05

### 3.4 Haptoglobin Phenotype Distribution

Frequencies of Hp phenotypes, Hp1 allele, and Hp 2 allele were in Hardy-Weinberg equilibrium (p> 0.05) for patients and control subjects. For Saudi type 2 diabetics, the Hp 1 allele frequency was .513 and for Hp2 allele was .487; while for control subjects it was .534 and .467 for Hp 1 allele and Hp2 allele respectively. Hp 2-1 was the most frequent phenotype among Saudi type 2 diabetics as well as healthy subjects (62.5% and 56.7% respectively). Data illustrated in Table 5.

**Table 5:** Distribution of Hp phenotype and Hp alleles frequency (Hp1 allele & Hp2 allele) in Saudi types 2 diabetics and in healthy nondiabetic subjects. Hp phenotypes expressed as number (%)

Group	Hp phenotype			Hp allele frequency		χ^2^ – test	*p*-value

	Hp 1-1	Hp 2-1	Hp 2-2	Hp1 allele frequency	Hp2 allele frequency		
Patients (n=60)	14(23.3)	34(56.7)	12(20.0)	.513	.487	5.006	.082
Controls (n=60)	16(26.7)	31(51.7)	13(21.6)	.534	.467	1.0763	.584

Abbreviations: Hp: haptoglobin; χ^2^: chi-square

### 3.5 Serum Levels of Inflammatory Markers in Relation to Hp Phenotypes

Table 6 demonstrates the relationship between Hp phenotypes (Hp 1-1, Hp 2-1, Hp 2-2) and serum concentrations of IL-6 (ng/mL), *hs*-CRP (mg/L), Hp (mg/dL) in type 2 diabetic patients and control subjects. This study couldn`t reveal any significant differences (p > 0.05) between the Hp phenotypes and any of these inflammatory markers.

**Table 6:** Serum levels of inflammatory markers in relation to Hp phenotypes in type 2 diabetics and healthy non-diabetic subjects

	patients			healthy non-diabetic subjects		

	Hp1-1	Hp2-1	Hp2-2	Hp1-1	Hp2-1	Hp2-2
Hp (mg/dl)	116.2±15.0	123.4±14.2	123.2±14.2	83.04±9.2	84.6±15.3	85.94±8.0
		P= 0.313	P=.402		P=.775	P=.519
*hs*-CRP (mg/l)	1.84±0.4	2.07±.0.14	2.19±.0.3	1.55± .0.4	1.71±0.7	1.94±0.9
		P=.525	P=.595		P=.471	P=.240
IL-6 (ng/ml)	7.46±1.7	7.80±1.1	7.84±1.1	4.72±1.8	5.06±1.7	4.96±1.9
		P=.624	P=.629		P= .640	P=.805

Data expressed as mean ±SD No significant difference observed when comparing Hp2-1 vs. Hp 1-1 OR Hp2-2 vs. Hp 1-1 (p-value > 0.05) in patients and healthy nondiabetic subjects

## 4. Discussion

During the last few years, the role of inflammatory factors in type 2 diabetes has become focus of great interest (Ebeling et al., 2001; Greenfield & Campbell, 2006; Donath & Shoelson, 2011). In this report, we have shown significantly higher serum concentrations of inflammatory markers (IL-6, *hs*-CRP, and Hp) in type 2 diabetics compared to controls. Our finding is in agreement with recently published reports which documented strong associations between type 2 diabetes and the mentioned acute phase proteins (Campenhout et al., 2006; Mirza et al., 2012). These findings may support the hypothesis that individuals who progress to type 2 diabetes show features of low-grade inflammation years in advance of disease onset which has been proposed to be involved in the pathogenetic processes causing the disease (Kristiansen & Mandrup-Poulsen, 2005). Interestingly, insulin itself is an inhibitor of acute-phase protein synthesis and in animal models of diabetes; the acute-phase response is increased by insulin deficiency indicating that there could be a positive feedback whereby cytokine-induced insulin resistance further augments the acute-phase response (Pickup 2004). Moreover, increasing evidence indicates that chronic mild inflammation linked to obesity is closely associated with the development of IR (Ouchi & Walsh, 2007). On the other hand, IR is strongly associated with obesity and is believed to precede the impairment in insulin secretion (Libman & Arslanian, 2007). This study demonstrated a significant association between the investigated inflammatory markers (Hp & *hs*-CRP) and body mass index as a marker of obesity. In consistent with our finding, it has been positively correlated human obesity with blood levels of CRP (Deepa et al., 2006) and Hp (Chiellini et al., 2004; Engstrom et al., 2004). The mechanisms for the significant associations between these acute phase proteins and obesity are unclear, but several explanations are possible. Subcutaneous and intra-abdominal adipose tissue is a major source of IL-6 production, which in turn stimulates the liver synthesis of C-reactive protein and haptoglobin (Pickup, 2004; Samara et al., 2008). Another explanation considered adipose tissues as active endocrine tissues which produce haptoglobin as well as other adipokines like IL-6 (Doumatey et al., 2010). The latter explanation can be supported by the findings of Fain et al (2004) who demonstrated a direct proportional release of Hp with IL-6 by explants of human visceral and subcutaneous adipose tissue in primary culture. Moreover, consistent with previous reports (Kado et al., 1999; Rodrı´guez-Mora´n et al., 1999; Tan et al., 2004; Suzuki et al., 2009), data demonstrates positive correlations between serum concentration of the examined inflammatory markers and selected variables (serum glucose and HbA1c).

Several studies have related Hp polymorphism to susceptibility and outcome in important diseases including diabetes (Wobeto et al., 2007; Koda et al., 2008; Costacou et al., 2008). However, this association between the Hp gene and diabetes varies between populations (Quaye et al., 2006). In this study data showed that the Hp1 allele frequencies among Saudi diabetics and healthy subjects were 0.513 and 0.534 respectively. In addition, it is documented that the Hp 2-1 was the most frequent Hp phenotype among Saudi type 2 diabetics. In contrast to our finding, Hp 2-2 was determined as the most common polymorphism in European diabetic population (Asleh et al., 2008). This may be attributed to the dramatic changes in the Saudi population lifestyle which accelerate the onset of type 2 diabetes since Hp 2-1 was the predominant Hp phenotype as reported by Awadallah et al (2001) for a Saudi sample population (n= 1002) in Southern Saudi Arabia.

It has been reported an association of Hp concentration with its phenotypes (Kasvosve et al., 2000). However, in our study, serum Hp concentrations among patients and control subjects were free of phenotypic-dependence. Moreover, data did not show any significant associations between *hs*-CRP /IL-6 and Hp phenotypes as reported by Bessa et al. (2007). The likely explanation regarding *hs*-CRP could be related to genetic variations of CRP genes and stimulants (such as obesity and low levels of physical activity) which may require different mechanisms. Also it was reported that CRP levels were age dependent increasing gradually to very old age to approximately 3 mg/L (Kluft et al., 2003). Further research is needed to elucidate the potential mechanism underlying the association between haptoglobin phenotypes and changes in CRP and IL-6 levels.

## 5. Conclusion

The major findings observed in the present study conducted in Saudi populations as follows: (i) Serum levels of IL-6, *hs*-CRP, and Hp were significantly increased in type 2 diabetics, (ii) obesity (measured by BMI) was associated with inflammatory markers *hs*-CRP and Hp in the diabetic patients, (iii) Serum concentration of the examined inflammatory markers was positively correlated with blood glucose and HbA1c, (iv) The Hp 2-1 phenotype was the predominant phenotype among Saudi both type 2 diabetics and healthy subjects, (v) there was no association documented between the Hp phenotypes and serum concentrations of the three aforementioned inflammatory biomarkers.
